# Antioxidant Capacity and Therapeutic Applications of Honey: Health Benefits, Antimicrobial Activity and Food Processing Roles

**DOI:** 10.3390/antiox14080959

**Published:** 2025-08-04

**Authors:** Ivana Tlak Gajger, Showket Ahmad Dar, Mohamed Morsi M. Ahmed, Magda M. Aly, Josipa Vlainić

**Affiliations:** 1 Department for Biology and Pathology of Fish and Bees, Faculty of Veterinary Medicine, University of Zagreb, 10000 Zagreb, Croatia; 2 Division of Agricultural Entomology, Sher-e-Kashmir University of Agricultural Sciences and Technology of Kashmir, Srinagar 191111, India; drshowketshameem@gmail.com; 3 Department of Biological Sciences, Faculty of Science, King Abdulaziz University, Jeddah 21589, Saudi Arabia or mmmahmed6@gmail.com (M.M.M.A.); mmmohammad@kau.edu.sa (M.M.A.); 4 Nucleic Acids Research Department, Genetic Engineering and Biotechnology Research Institute (GEBRI), City for Scientific Research and Technological Applications, Alexandria 21934, Egypt; 5 Laboratory for Oxidative Stress, Institute Ruđer Bošković, Bijenička Cesta 54, 10000 Zagreb, Croatia; josipa.vlainic@irb.hr

**Keywords:** *Apis mellifera*, reactive oxygen species, honey, diseases, health disorders, antioxidants, phytochemicals

## Abstract

Honey is a natural product of honeybees that has been consumed for centuries due to its nutritional value and potential health benefits. Recent scientific research has focused on its antioxidant capacity, which is linked to a variety of bioactive compounds such as phenolic acids, enzymes (e.g., glucose oxidase, catalase), flavonoids, ascorbic acid, carotenoids, amino acids, and proteins. Together, these components work synergistically to neutralize free radicals, regulate antioxidant enzyme activity, and reduce oxidative stress. This review decisively outlines the antioxidant effects of honey and presents compelling clinical and experimental evidence supporting its critical role in preventing diseases associated with oxidative stress. Honey stands out for its extensive health benefits, which include robust protection against cardiovascular issues, notable anticancer and anti-inflammatory effects, enhanced glycemic control in diabetes, immune modulation, neuroprotection, and effective wound healing. As a recognized functional food and dietary supplement, honey is essential for the prevention and adjunct treatment of chronic diseases. However, it faces challenges due to variations in composition linked to climatic conditions, geographical and floral sources, as well as hive management practices. The limited number of large-scale clinical trials further underscores the need for more research. Future studies must focus on elucidating honey’s antioxidant mechanisms, standardizing its bioactive compounds, and examining its synergistic effects with other natural antioxidants to fully harness its potential.

## 1. Introduction

**Figure 1:**
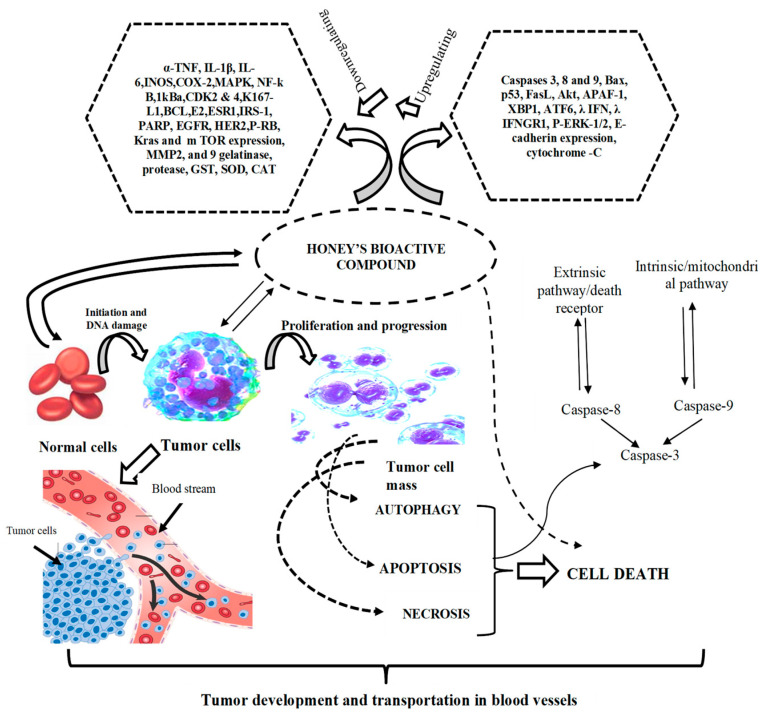
The role of bioactive compounds found in honey serves as a potential antitumor agent, inhibiting the transport of cancer cells and the phases of cell death (adapted from [28]).

Honey, a natural product processed by honeybees from the nectar or excretions of plant-sucking insects, has been a staple in human diets and traditional medicine for centuries. Beyond its culinary applications, honey is increasingly recognized for its therapeutic potential, particularly its antimicrobial, anti-inflammatory, and antioxidant properties [1]. In recent decades, scientific attention has turned toward the antioxidant capacity of honey, driven by the rising global occurrence of oxidative stress-related diseases such as cardiovascular disorders, diabetes, cancer, and neurodegenerative conditions [2]. Oxidative stress is an imbalance between reactive oxygen species (ROS) and the body’s endogenous antioxidant defenses, leading to cellular damage, inflammation, and disease progression [3]. Antioxidants are crucial in neutralizing ROS and include enzymatic compounds such as glutathione, superoxide dismutase, mycothiol, and bacillithiol, which collectively protect cellular structures and functions from oxidative degradation [4,5]. At the same time, exogenous antioxidants from natural sources, including honey, play a significant complementary function in preventing oxidative damage and supporting overall health [6,7,8]. Honey contains over 200 bioactive compounds, including sugars, enzymes, amino acids, organic acids, vitamins, minerals, and a rich profile of antioxidants [9,10]. Among these, key antioxidant constituents include phenolic acids, flavonoids, ascorbic acid, catalase, glucose oxidase, and carotenoids, all of which act through various mechanisms such as hydrogen atom donation, radical scavenging, and modulation of redox-sensitive signaling pathways [11,12,13,14]. Notably, the antioxidant action of honey arises not only from individual components but also from their synergistic interactions, which enhance their overall bioactivity. These compounds may contribute to the regulation of vital physiological processes: thermogenesis, vasodilation, collagen synthesis, and immune modulation [13,15]. Honey’s chemical composition and antioxidant potential are highly variable, influenced by its botanical origin, geographic region, seasonal factors, and beekeeping practices, including processing and temperature control [16]. This complexity poses challenges for standardization and clinical application, although it also provides a broad matrix of therapeutic opportunities (Figure 1). β-carotene and vitamins C and E, which are trace compounds present in honey, further contribute to its health benefits by reducing oxidative stress, improving cardiovascular function, preventing strokes, and exerting anti-carcinogenic effects through the inhibition of tumor necrosis factor [17,18].

Current research has clarified honey’s ability to influence signaling pathways related to oxidative stress and inflammation, such as nuclear factor kappa B (NF-κB) and mitogen-activated protein kinases (MAPKs), offering mechanistic insight into its therapeutic actions [19]. Both laboratory-controlled and in vivo studies have supported honey’s role in reducing oxidative biomarkers and lipid peroxidation, regulating blood glucose levels, and protecting against cellular apoptosis and DNA damage [20,21]. Furthermore, honey has demonstrated potential in enhancing immune responses by promoting the activity of immune cells and exerting antimicrobial effects [2]. Emerging research also highlights honey’s neuroprotective capabilities, indicating its potential role in mitigating age-related cognitive decline and neurodegenerative diseases [22]. Despite these promising findings, gaps remain in fully understanding honey’s antioxidant mechanisms and its clinical applicability. There is a critical need for standardized analytical protocols and large-scale, randomized clinical trials to validate honey’s therapeutic potential and determine the optimal dosages and formulations [23]. While the historical and ethnopharmacological uses of honey are well-documented [24], its integration into modern healthcare as a scientifically supported antioxidant therapy remains underdeveloped.

In this review, we aim to present a comprehensive analysis of the antioxidant properties of honey, its chemical composition, mechanisms of action, and health benefits. We also discuss the challenges related to standardization and quality assessment, while identifying current research gaps and future directions. By synthesizing recent advances, this work seeks to deepen our understanding of honey as a potent natural antioxidant with significant implications for preventing and managing diseases in humans related to oxidative stress [25,26,27]. While writing, a structured literature search protocol was followed, which included defined search strategies, inclusion and exclusion criteria, and methods for data extraction to ensure the relevance and quality of the studies reviewed.

## 2. Chemical Composition and Its Properties

Honey is a complex natural product made up of a variety of bioactive molecules, whose composition is largely influenced by floral origin, environmental conditions, and beekeepers’ management practices and processing methods. The most important bioactive constituents are phytochemicals, natural compounds derived from plant sources, which have demonstrated significant health-promoting potential, including antioxidant, antimicrobial, and anti-inflammatory activities [29]. A major class of phytochemicals present in honey is polyphenols, which are well-recognized for their antioxidant capacity [30]. The polyphenolic profile of honey includes a range of flavonoids and phenolic acids such as 4-hydroxybenzoic acid, p-coumaric acid, vanillic acid, caffeic acid, syringic acid, gallic acid, quercetin, kaempferol, myricetin, pinobanksin, pinocembrin, and chrysin. Additional phenolic acids commonly found include hesperetin, ellagic acid, ferulic acid, and chlorogenic acid [31,32]. The concentrations of these compounds are highly variable and are strongly influenced by local forage conditions [33,34]. As such, the polyphenolic and phenolic acid content serve not only as a functional health marker but also as a chemotaxonomic index of botanical origin in monofloral honeys [33].

In addition to polyphenols, volatile organic compounds (VOCs) contribute to both the enchanting therapeutic and sensory qualities of honey. These include terpenes (e.g., linalool, linalool oxide isomers, lilac alcohol and aldehyde isomers), norisoprenoids (e.g., isophorone, vomifoliol), and aromatic alcohols such as benzyl alcohol and 2-phenylethanol. Other notable VOCs include benzaldehyde, furfural, acetic acid, cis-linalool oxide, and 3,5-dimethoxy-4-hydroxybenzoic acid methyl ester (DMHBME), as well as veratric acid, syringic acid, eudesmic acid, and trimethoxybenzoic acid derivatives [35]. These substances enhance both the antimicrobial and antioxidant properties of honey, as well as its intricate aroma and flavor.

The color and mineral composition of honey are also important indicators of its quality and bioactivity. Darker honeys, such as those from buckwheat, chestnut, wildflower sources, or honeydew, tend to have higher concentrations of antioxidants and minerals. This correlates with a more intense flavor profile and enhanced health benefits [36,37,38,39]. Stingless bees, in particular, produce honey with distinct colorations depending on the nectar source, ranging from water white to dark amber hues [40]. From a macronutrient perspective, honey primarily consists of sugars (79.6–80%) and water (17.2–18%). The main sugars are fructose (38.19–40%) and glucose (31.28–35%), with smaller amounts of sucrose (1.3–5%) and maltose (7.3–10%) [41]. These sugars play a role not only in honey’s sweetness and preservation but also in modulating its antioxidant potential via Maillard reaction products during processing. Honey’s minor constituents, though present in small quantities, play a disproportionately large role in its therapeutic efficacy. These include organic acids (0.57–1.0%), proteins (0.266–0.50%), nitrogenous compounds (0.043%), and amino acids (0.05–0.1%) [36]. Trace elements and minerals (0.04–0.17%) are essential for enzymatic and redox functions [42,43]. In addition, honey contains a variety of micronutrients and bioactive compounds, including carotenoids, flavonoids, vitamins (C, B-complex, K, and E), volatile compounds (e.g., aldehydes, alcohols, esters, ketones, and phenols), sugar alcohols, and colloids, which collectively account for approximately 2.1% of its total biochemical profile [41]. These constituents are essential not only for antioxidant activity but also for wound healing, immunomodulation, and other biological effects. To summarize, honey’s chemical composition is rich and diverse, encompassing a wide spectrum of nutritional, sensory, and bioactive compounds (Table 1). This chemical complexity underlies honey’s broad pharmacological potential, making it a unique functional food and a promising candidate for further investigation into the prevention and therapy of antioxidant-related diseases.

## 3. Antioxidant Capacity of Honey and Its Health Benefits

Oxygen plays a critical role in cellular respiration and energy production; however, its metabolic byproducts, particularly ROS, contribute to oxidative stress, aging, and a wide range of degenerative diseases [44]. ROS and free radicals, as unstable molecules with unpaired electrons, are produced during normal cellular metabolism and exert damaging effects on lipids, proteins, and nucleic acids through complex initiation, propagation, and termination reactions [45,46]. The accumulation of oxidative damage over time accelerates aging and contributes to the “growth” of chronic conditions such as cancer, cardiovascular disorders, and neurodegenerative diseases. Antioxidants counteract oxidative stress by neutralizing free radicals and maintaining redox homeostasis. These compounds may be produced endogenously or obtained from external sources, primarily the diet [47,48,49]. Among natural dietary sources, honey has received considerable attention due to its rich content of bioactive compounds that exhibit potent antioxidant properties [50]. Honey contains a diverse array of antioxidants, including ascorbic acid, catalase, glucose oxidase, flavonoids, phenolic acids, carotenoids, organic acids, amino acids, proteins, and Maillard reaction products [51]. For example, vitamin C enhances cardiovascular and immune function, while phenolic compounds such as caffeic acid, quercetin, and chrysin exert antioxidant activity through mechanisms including electron donation, hydrogen atom transfer, metal ion chelation, and scavenging of ROS [52].

**Figure 2:**
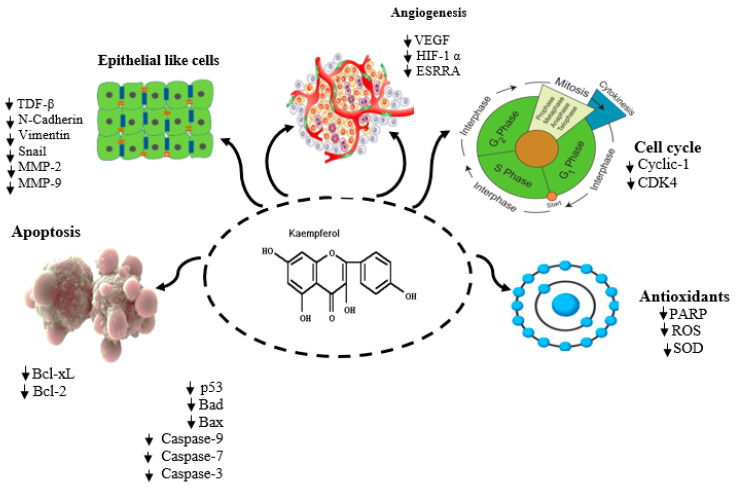
Illustration of the antioxidant mechanism of kaempferol and its role in cancer treatment, along with a schematic representation of the pathways and targets affected by kaempferol identified in vitro cancer experiments (adapted from [53]). Arrows show the sequential steps of the reaction/process.

Polyphenols such as apigenin, kaempferol, pinocembrin, galangin, chrysin, quercetin, and acacetin are widely identified in honey and contribute significantly to its pharmacological effects (e.g., Figure 2), including antioxidant, anti-inflammatory, and chemo-preventive actions [53]. The antioxidant properties of honey are also highly dependent on its post-harvest processing and storage conditions. Generally, darker honeys exhibit greater antioxidant activity. Buckwheat honey, for instance, possesses antioxidant potential comparable to 1 mM α-tocopherol in lipid peroxidation assays and exceeds the antioxidant content of various fruits and vegetables [54]. Specific honey varieties such as sage and buckwheat honey exhibit high antioxidant activity, with values reported at 21.3 × 10^−5^ and 4.32 × 10^−3^ equivalents, respectively [55].

The phytochemical composition of honey also includes bioactive phenolics like quercetin, kaempferol, pinobanksin, and caffeic acid, which not only enhance antioxidant activity but also serve as chemical indicators that reveal the botanical origin of honey [53]. Additionally, honey contains antimicrobial phytochemicals such as terpenes, benzaldehyde, 2-phenylethanol, and volatile aldehydes that synergize with antioxidant compounds to provide broad-spectrum biological effects [56,57]. Although mentioned minor components represent only about 2.1% of the total honey composition, they include bioactive molecules such as flavonoids, carotenoids, sugar alcohols, and aromatic compounds that are central to honey’s antioxidant efficacy. Both in vitro and in vivo studies have substantiated the antioxidant effects of honey. In vitro assays have demonstrated that honey-derived polyphenols possess high oxygen radical absorbance capacity (ORAC) and effectively inhibit lipid peroxidation and oxidative damage to cellular macromolecules [53]. Buckwheat honey has been shown to protect endothelial cell lines (EA.hy926) from oxidative stress induced by cumene hydroperoxide (CuOOH), hydrogen peroxide (H_2_O_2_), and peroxyl radicals (AAPH), primarily through glutathione (GSH) restoration, inhibition of ROS generation, and prevention of lipid peroxidation [58,59]. These protective effects can be translated into physiological benefits in humans. For instance, individuals consuming 1.5 g/kg of body weight of buckwheat honey exhibited significantly elevated serum antioxidant levels compared to those ingesting corn syrup [60]. Another clinical study reported increases in vitamin C, β-carotene, uric acid, and glutathione reductase by 47%, 3.0%, 12%, and 7%, respectively, following daily honey consumption [25]. The bioavailability of phenolic compounds in honey was confirmed by HPLC-MS analysis [61]. The antioxidant effects of honey are not only the result of individual components but also of synergistic interactions among polyphenols and other bioactive molecules, which enhance their stability, bioefficacy, and reactivity [59]. Even processed honey has demonstrated the capacity to elevate plasma antioxidant levels, suggesting therapeutic relevance for chronic disease prevention and health maintenance [62]. Considering that the average person consumes a few kg of sweeteners annually, replacing refined sugars with honey offers a natural and functional approach to increasing antioxidant intake and reducing oxidative stress. The evidence supports the view that honey, as a complex natural honeybee product, has significant potential as a dietary antioxidant with protective effects against ROS-mediated cellular damage and disease progression.

## 4. Role of Antioxidants in Preventing Cancer

**Figure 3:**
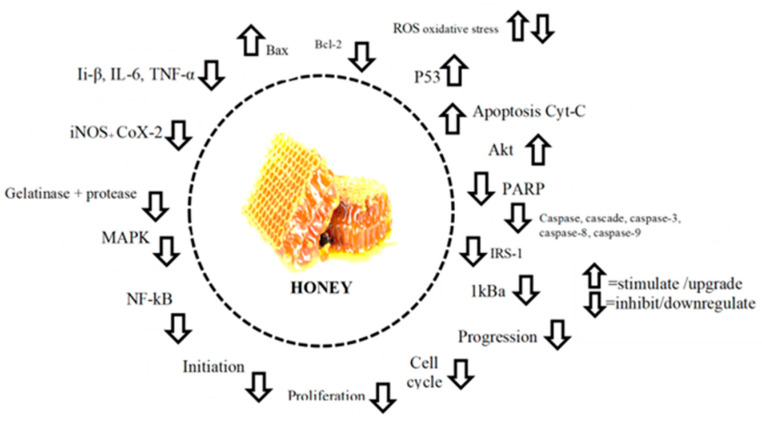
Molecular target modulation as the anticancer effects of honey (adopted from [63]). Legend: Bcl-2 = B cell lymphoma 2; Bcl-xL = B cell lymphoma extra-large; Cyt. C = cytochrome C; MAPK = mitogen-activated protein kinase; NF-κB = nuclear factor kappa B; Akt = altered PI3 kinase; IRS-1 = insulin receptor substrate; IL = interleukin; COX-2 = cyclooxygenase 2; TNF-α = tumor necrosis factor alpha; iNOS = inducible nitric oxide synthase; IκBα = inhibitor of kappa B; PARP = poly ADP-ribose polymerase.

The antioxidant constituents of honey protect against oxidative damage and contribute significantly to the prevention and potential treatment of cancer (Figure 3). Honey-derived polyphenols have been shown to interfere with key steps in tumorigenesis, including oxidative stress modulation, suppression of angiogenesis, and induction of programmed cell death in malignant cells. Among the most studied bioactive compounds present in honey are flavonoids and phenolic acids such as quercetin, galangin, chrysin, kaempferol, apigenin, acacetin, pinocembrin, artepillin C, and caffeic acid phenethyl ester (CAPE). These molecules display strong radical-scavenging activity and have demonstrated substantial anticancer potential in both in vitro and in vivo models [63,64]. Through standard antioxidant assays including DPPH and FRAP, the capacity of these compounds to counteract oxidative stress has been well characterized, suggesting a strong link between their antioxidant properties and their cytotoxic effects on cancer cells.

**Figure 4:**
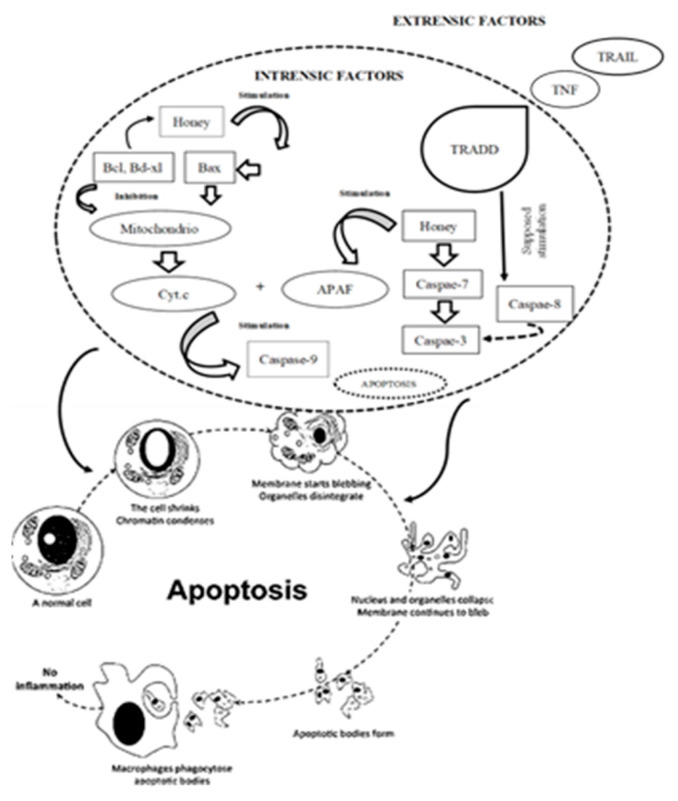
Effect of honey on apoptotic pathways (adopted from [6,63]). Legend: Bcl-2 = B cell lymphoma 2; Bcl-xL = B cell lymphoma extra-large; Cyt. C = cytochrome C; APAF-1 = apoptotic protease-activating factor 1; TNF = tumor necrosis factor; TRAIL = TNF-related apoptosis-inducing ligand; TRADD = TNFR-associated death domain protein.

In addition to redox modulation, the antiangiogenic properties of honey polyphenols have gained increasing attention. Using endothelial cell models, Wahid et al. (2024) demonstrated that compounds such as quercetin, caffeine, and phenethyl ester significantly impaired angiogenic activity by reducing endothelial proliferation and tube formation [65]. Artepillin C, galangin, and kaempferol were also found to inhibit neovascularization, albeit with a somewhat reduced potency [66]. Interestingly, polyphenols with only moderate antioxidant scores, such as apigenin and acacetin, were still capable of disrupting endothelial morphogenesis, suggesting that antiangiogenic activity may occur through mechanisms distinct from direct antioxidant action. These findings have prompted proposals to explore honey polyphenols as potential leads for the development of antiangiogenic drugs. Alarjani et al. [67] and Mahani et al. [68] emphasized the therapeutic relevance of these natural compounds in oncology. At the same time, Zulkifli et al. further highlighted their pharmaceutical applicability, particularly in the context of multi-targeted chemoprevention strategies and sleep [69]. A synergistic role of honey-derived compounds with other bioactive substances has also been suggested. For instance, Qu et al. demonstrated that co-administration of caffeine with honey antioxidants significantly suppressed colon tumorigenesis in rats, despite caffeine’s previously reported oncogenic potential when isolated [70]. This finding underscores the importance of matrix effects and compound interactions within the honey phytochemical profile. Several honey constituents act directly on tumor cells by activating intrinsic apoptotic pathways (Figure 4). CAPE, for example, has been shown to exert antimitogenic, cytostatic, and immunomodulatory effects, further supporting its utility in anticancer applications [71].

**Figure 5:**
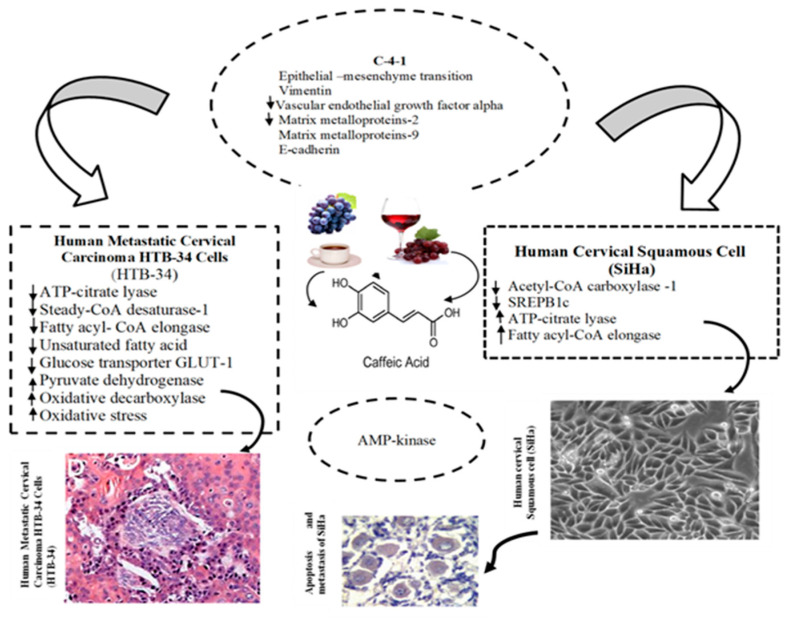
Illustration of the effects of caffeic acids upregulating the expression of enzymes involved in the production of fatty acids, followed by AMPK expression in cervical cancer cells (adapted from [80]). Arrows show the sequential steps of the reaction/process.

Chrysin induces apoptosis through proteasome inhibition and activation of the p38-MAPK signaling cascade, leading to upregulation of p21^Waf1/Cip1 in glioma cells [72,73], while also modulating the Akt pathway and enhancing caspase-3 activation in leukemia models [74]. Galangin has demonstrated selective cytotoxicity in HL-60 leukemia cells, where it induces DNA fragmentation without disrupting membrane integrity, indicative of programmed cell death [64]. Quercetin, one of the most extensively studied honey polyphenols, has shown the ability to inhibit various kinases involved in cancer cell signaling, including membrane tyrosine kinases and protein kinase C. At lower concentrations, quercetin can paradoxically promote proliferation in A549 lung carcinoma cells, while higher doses induce apoptosis and cell cycle arrest [75]. Furthermore, Carrillo-Martinez et al. (2024) noted that quercetin and structurally related flavonoids enhance Fas-ligand and p53 expression, which are crucial regulators of apoptosis in cancer cells such as A549, HepG2, and H460 [76]. Kaempferol has been reported to trigger mitochondrial apoptosis by elevating ATP production and enhancing the expression of apoptosis-inducing factor (AIF) and caspase-3 in H460 cells [77], and by inducing cell cycle arrest in leukemia cell lines [78]. Pinocembrin disrupts mitochondrial membrane potential in HCT-116 colon cancer cells, promoting cytochrome c release and downstream caspase activation [79]. Similarly, apigenin has demonstrated cytotoxic effects in a range of tumor types, including hepatic, breast, cervical, and neuroblastoma cells, through mechanisms involving DNA damage, mitochondrial dysfunction, and caspase-mediated apoptosis. Taken together, these findings support the concept that honey contains a complex matrix of polyphenolic compounds capable of exerting multifaceted anticancer effects (e.g., Figure 5). By acting at the intersection of oxidative stress modulation, angiogenesis inhibition, and apoptotic signaling, these compounds provide a compelling rationale for further exploration in both preventive and therapeutic oncology settings.

## 5. Antioxidants and Cardiovascular Diseases

The role of antioxidants in the prevention and management of cardiovascular diseases (CVDs) has been extensively documented, particularly regarding natural sources such as honey. Honey possesses a rich array of enzymatic and non-enzymatic antioxidants, including polyphenols, flavonoids, ascorbic acid (vitamin C), and reduced glutathione, which collectively contribute to its significant cardioprotective properties [81]. These antioxidants play a crucial role in neutralizing ROS, which are implicated in the pathogenesis of various cardiovascular conditions like myocardial infarction, ischemia-reperfusion injury, atherosclerosis, and hypertension [82]. Experimental studies using animal models have provided strong evidence that honey consumption offers cardiovascular benefits. In rats anesthetized with urethane, pre-treatment with honey (5 g/kg) one hour before administration of epinephrine (100 µg/kg) significantly mitigated hyperadrenergic-induced cardiovascular dysfunction. Improvements were observed in electrocardiographic parameters, vasomotor function, and inotropic and chronotropic responses, particularly under conditions simulating anaphylaxis, cardiac arrest, asthma, and hemorrhage [83,84]. These effects are attributed to the high total antioxidant capacity (TAC) of honey, particularly due to its elevated levels of SOD and reduced glutathione, despite lower catalase activity [85].

The cardioprotective potential of honey extends beyond its radical-scavenging activity. Polyphenolic compounds in honey, including quercetin, caffeic acid phenethyl ester (CAPE), kaempferol, galangin, chrysin, and pinocembrin, have been shown to modulate multiple redox-sensitive signaling pathways. These include enhancing nitric oxide (NO) bioavailability, suppressing superoxide generation, and preserving endothelial integrity [86,87]. For instance, kaempferol and galangin have stabilized mitochondrial function, reduced cardiomyocyte apoptosis, and maintained cardiac tissue structure following ischemic insults [88]. Additionally, clinical studies indicate that moderate antioxidant supplementation significantly enhances cardiovascular health by reducing oxidative stress biomarkers such as malondialdehyde (MDA) and conjugated dienes (CD), and increasing overall plasma antioxidant capacity [89]. In one controlled study involving myocardial infarction survivors and healthy middle-aged men, daily supplementation with 100 mg of vitamin E, 100 mg of vitamin C, 6 mg of β-carotene, and 50 µg of selenium led to measurable improvements in antioxidant status and lipid peroxidation markers [90,91]. These outcomes support the inclusion of antioxidant-rich foods such as honey as adjunctive therapy in cardiovascular care, particularly in populations at elevated risk. Furthermore, honey can influence lipid metabolism. For example, studies in animal models of hypertension and hyperlipidemia showed that honey administration reduced total cholesterol and LDL levels while increasing HDL concentrations. These effects are mediated by upregulation of endogenous antioxidant enzymes, including SOD, CAT, and GPx, and by reducing lipid peroxidation [92,93,94].

From a vascular health perspective, honey enhances endothelial function through NO-mediated vasodilation. This is facilitated by the presence of minerals such as magnesium, sodium, and chlorine, and is potentiated by the antioxidant activity of vitamin C [95]. Flavonoids, such as apigenin and pinocembrin, further contribute to this effect by inhibiting platelet aggregation through the modulation of thromboxane A2 and the enhancement of prostacyclin synthesis, thereby reducing the risk for thrombosis [96,97]. In a prospective cohort study involving 34,492 postmenopausal women, higher flavonoid intake, including from honey, was associated with a significantly reduced risk of coronary artery disease (CHD) [98]. Adjusted risk assessments indicated a downward trend in CHD-related deaths across increasing quintiles of flavonoid consumption, with broccoli and other flavonoid-rich foods contributing substantially to this effect. The benefits of honey are also evident in surgical and pediatric cardiovascular contexts. Elevated levels of endothelin-1, a potent vasoconstrictor linked to hypertension and heart failure, are attenuated by honey polyphenols such as quercetin and kaempferol. This has particular relevance in postoperative care following cardiopulmonary bypass, where antioxidant therapies, such as Salvia miltiorrhiza injections rich in phenolic compounds, have demonstrated effectiveness in reducing endothelin-1 levels [99]. In summary, honey’s diverse antioxidant components confer multifaceted cardiovascular benefits. These include reducing oxidative stress, enhancing endothelial and mitochondrial function, improving lipid profiles, regulating inflammatory responses, and modulating key signaling pathways involved in vascular homeostasis and cardiac protection. Given its natural origin and broad therapeutic range, honey emerges as a promising adjunct in both the prevention and management of cardiovascular diseases.

### 5.1. Quercetin

Quercetin, a widely studied flavonoid found in apples, onions, green tea, and honey, exerts cardiovascular protective effects by inhibiting vascular smooth muscle cell (VSMC) hypertrophy and reducing oxidative stress and inflammation. Angiotensin II (Ang-II)-induced VSMC hypertrophy involves activation of MAPK pathways such as JNK, ERK1/2, and p38. Also, quercetin selectively inhibits Ang-II-induced JNK activation, attenuating hypertrophic signaling and apoptosis in VSMCs without affecting ERK1/2 and p38 [100,101,102]. Also, quercetin inhibits tyrosine phosphorylation of adaptor proteins such as Shc and blocks PI3-K/Akt pathway activation, which are key drivers of VSMC hypertrophy [103]. The quercetin analog LY294002 also inhibits Akt phosphorylation, corroborating this pathway’s significance [104]. In vivo studies have demonstrated that quercetin lowers blood pressure and improves endothelial function in spontaneously hypertensive rats (SHR) by activating eNOS and suppressing NADPH oxidase [105]. Clinical trials reveal quercetin’s antihypertensive effects in stage-1 hypertension, though not in prehypertension. The mode of quercetin administration critically affects its bioavailability and efficacy, with oral gavage providing superior cardiovascular benefits compared to dietary supplementation [106,107]. Quercetin’s pleiotropic effects, including antioxidant, anti-inflammatory, and anti-proliferative actions, underpin its potential as a cardioprotective agent [108].

### 5.2. Acacetin

Acacetin, a natural flavone, acts as a selective inhibitor of atrial-specific potassium currents, particularly K+ current (IKur), making it a promising agent for atrial fibrillation treatments [109]. Unlike many antiarrhythmics that prolong the QT interval and risk ventricular arrhythmias, acacetin prolongs atrial action potential duration and refractory period without significant ventricular effects [110,111]. In anesthetized dogs, intraduodenal acacetin effectively suppressed atrial fibrillation without QT prolongation or adverse ventricular effects, unlike non-selective agents such as sotalol [112]. This atrial selectivity stems from acacetin’s targeted blockade of atrial ion channels and calcium-activated SK channels [109,113].

### 5.3. Caffeic Acid Phenethyl Ester (CAPE)

Caffeic acid phenethyl ester (CAPE), a bioactive component of honeybee propolis, exerts potent antioxidant and anti-inflammatory effects relevant to cardiovascular health. One of CAPE’s primary molecular actions is the inhibition of NF-κB signaling, a central pathway in oxidative stress and vascular inflammation [114]. Additionally, CAPE suppresses both NF-κB and JNK signaling cascades, which have been shown to improve insulin sensitivity and reduce inflammatory responses in diabetic animal models [115]. These findings highlight CAPE’s therapeutic potential in modulating intracellular inflammatory pathways associated with cardiometabolic disorders.

### 5.4. Kaempferol

Kaempferol, a flavonol abundant in various plants and honey, exhibits antioxidant, anti-inflammatory, and anti-platelet activities, contributing to its cardiovascular protective effects [108]. Early studies demonstrated kaempferol-induced vasorelaxation in isolated arteries, mediated via both endothelium-dependent nitric oxide and endothelium-independent mechanisms. More recent investigations reveal that kaempferol protects cardiac myocytes against ischemia-reperfusion (I/R) injury by modulating endoplasmic reticulum (ER) stress responses and apoptosis. It decreases the expression of ER stress markers such as GRP78 and CHOP and regulates the Bcl-2/Bax ratio, reducing cardiomyocyte apoptosis after I/R insult. This modulation of ER stress and apoptotic pathways represents a novel mechanism for kaempferol’s cardio protection role [116].

### 5.5. Galangin

Galangin (3,5,7-trihydroxyflavone), a naturally occurring flavonol found in honey and the rhizome of *Alpinia officinarum*, exhibits potent antioxidant and anti-inflammatory properties relevant to cardiovascular health. Recent studies demonstrate that galangin attenuates endothelial dysfunction by activating the heme oxygenase-1 (HO-1) signaling pathway, reducing oxidative stress, and suppressing proinflammatory mediators such as VCAM-1 and TNF-α in vascular endothelial cells [117]. In animal models of metabolic syndrome, galangin improves aortic function by restoring endothelial nitric oxide synthase (eNOS) activity, enhancing nitric oxide (NO) availability, and inhibiting the angiotensin II/AT1R/TGF-β signaling axis [118]. These findings highlight galangin’s therapeutic potential in preventing vascular damage associated with hypertension and cardiometabolic disorders.

## 6. Antioxidants and Neural Diseases

Flavonoids, a diverse class of polyphenolic compounds found in honey, various fruits, vegetables, and plant-based products, have been widely investigated for their pharmacological properties, including anti-inflammatory, anticancer, and antioxidant activities. However, their mechanisms in neuroprotection, particularly in the context of neurodegenerative disorders, are still under active investigation [119]. Neurodegenerative diseases such as Alzheimer’s and Parkinson’s are now increasingly understood to involve chronic neuroinflammation, largely mediated by the activation of microglia, the resident immune cells of the central nervous system (CNS). Microglial activation leads to the release of pro-inflammatory cytokines, NO, and ROS, contributing to neuronal injury and disease progression [120]. Flavonoids, including apigenin, have been shown to modulate this inflammatory microenvironment. In murine BV-2 microglia cells, apigenin suppresses LPS-induced activation by inhibiting nuclear factor kappa B (NF-κB) signaling and promoting nuclear translocation of Nrf2, a master regulator of antioxidant responses [121]. This dual action results in decreased expression of inducible nitric oxide synthase (iNOS) and cyclooxygenase-2 (COX-2), ultimately reducing NO and prostaglandin E2 (PGE2) levels. Furthermore, apigenin exerts neuroprotective effects in models of cerebral ischemia-reperfusion (I/R) injury, such as middle cerebral artery occlusion (MCAO). In both in vitro and in vivo models, it mitigates oxidative stress, reduces infarct volume, and limits neuronal apoptosis. This protection is mediated via attenuation of endoplasmic reticulum (ER) stress, modulation of Bcl-2 family proteins, and suppression of JNK and p38 MAPK phosphorylation, while sparing extracellular signal-regulated kinase (ERK) pathways [122]. Beyond its anti-inflammatory and antioxidant effects, apigenin also modulates central neurotransmission, contributing to its sedative, antidepressant, and anxiolytic properties. Behavioral studies have shown that apigenin reduces anxiety-like behavior and decreases locomotor activity in rodents, effects likely mediated by its interaction with GABA_A receptors [123]. Patch-clamp experiments in HEK293 cells expressing α1β2γ2 GABA_A receptor subunits have revealed that apigenin inhibits GABA-evoked chloride currents, albeit with less potency compared to classical benzodiazepines. This supports a modulatory, rather than agonistic, role at these receptor sites. Apigenin also affects excitatory neurotransmission. In cultured cortical neurons, it inhibits NMDA receptor-mediated currents while sparing AMPA receptor activity, suggesting a selective antagonism of the NMDA receptor complex. This is particularly relevant since excessive glutamate signaling and NMDA receptor activation contribute to excitotoxicity, a common mechanism in stroke and neurodegeneration. Apigenin’s inhibition of NR2 subunits of NMDA receptors offers a novel target for its neuroprotective profile. In animal models of aging and neuroinflammation, chronic apigenin administration reduces hippocampal microglial activation and improves synaptic integrity without causing overt sedation or cognitive impairment, making it a promising candidate for neurodegenerative disease prevention and adjunct therapy [123].

### Pinocembrin

Pinocembrin, a flavonoid primarily found in honey and propolis, is recognized for its broad pharmacological properties, including antioxidant, anti-inflammatory, and neuroprotective activities. Among the polyphenolic compounds in honeybee products, pinocembrin is one of the most abundant and biologically active, contributing significantly to the radical-scavenging capacity of honey and its neuroprotective properties [124]. Recent studies have demonstrated that pinocembrin crosses the blood–brain barrier and offers neuroprotection in models of cerebral ischemia/reperfusion (I/R) injury. In a rat middle cerebral artery occlusion (MCAO) model, administration of pinocembrin at the onset of reperfusion significantly reduced infarct volume, improved neurological deficit scores, and increased survival rates [125,126]. These effects are associated with its ability to reduce ROS, suppress neuronal (nNOS) and inducible nitric oxide synthase (iNOS) expression, and increase intracellular glutathione levels, thereby enhancing redox homeostasis [126].

Pinocembrin also modulates apoptotic pathways by downregulating caspase-3 activity, limiting PARP cleavage, and reducing neurite retraction and lactate dehydrogenase (LDH) release, ultimately supporting neuronal survival [126]. Additionally, in global cerebral ischemia and chronic cerebral hypoperfusion models, pinocembrin has been shown to preserve blood–brain barrier (BBB) integrity, reduce cerebral edema, and restore cerebral blood flow [127]. Mitochondrial protection is a key mechanism underlying these effects. Pinocembrin enhances mitochondrial respiratory chain complex I and III activities, stabilizes the mitochondrial membrane potential, reduces mitochondrial swelling, and limits mitochondrial ROS production [128]. Restoration of cytochrome oxidase expression further contributes to mitochondrial integrity and neuronal energy homeostasis. These molecular and functional effects translate into improved cognitive outcomes, as evidenced by enhanced performance in behavioral assays such as the Morris water maze [128]. Collectively, these findings underscore the potential of pinocembrin as a promising therapeutic candidate for neurovascular protection, particularly in the context of stroke and vascular cognitive impairment.

## 7. Antimicrobial Activities of Honey

Phenolic compounds extracted from both dark and light honey types possess notable antioxidant and antimicrobial activities. Their antioxidant capacity is typically evaluated by assays like DPPH free radical scavenging and ferric reducing antioxidant power (FRAP), reflecting their ability to donate electrons and neutralize reactive species [129]. Bioactive phenolics also exhibit robust in vitro antibacterial effects. Studies on honeys, including *Apis laboriosa* subspecies, have demonstrated significant inhibition of Gram-negative (*Escherichia coli*, *Salmonella enterica* serovar *Typhimurium*) and Gram-positive (*Staphylococcus aureus*, *Bacillus subtilis*) bacteria. The potency correlates strongly with total phenolic and flavonoid content [130]. The antimicrobial action of honey is multifactorial, arising from both peroxide and non-peroxide mechanisms. Hydrogen peroxide, generated enzymatically by glucose oxidase, is a primary antibacterial agent. However, catalase-insensitive phenolic constituents, such as caffeic and ferulic acids, also play a crucial role, particularly in dark honeys [1,131]. Further research confirms that isolated phenolic acids and flavonoids, major contributors found in honey, inhibit bacterial growth through membrane disruption, enzyme inactivation, and synergistic antioxidant activity [132]. These findings support the view that phenolic-rich honeys deliver enhanced antimicrobial efficacy, highlighting their therapeutic and food safety potential.

## 8. Wound Healing

**Figure 6:**
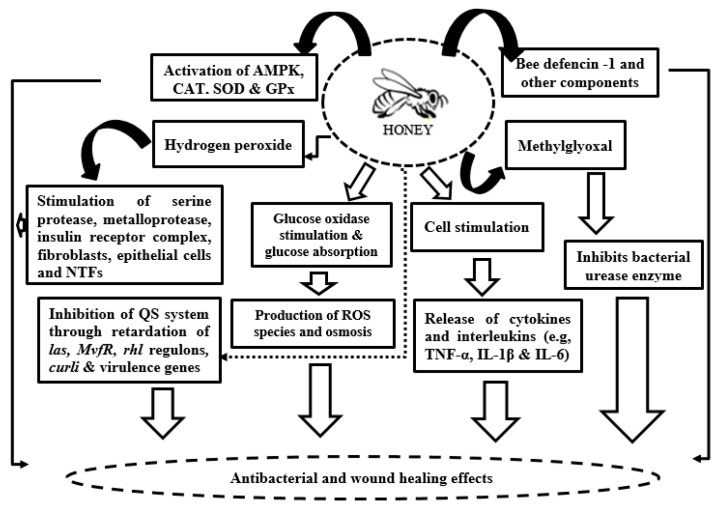
Mechanisms of antimicrobial and wound healing effects of honey (modified from [143]). Legend: AMPK = 5′adenosine monophosphate-activated protein kinase; QS = quorum sensing; SOD = superoxide dismutase; GPx = glutathione peroxidase; NTFs = nuclear transcription factors; TNF-α = tumor necrosis factor alpha; IL = interleukin.

Honey has increasingly attracted attention in clinical and regenerative medicine due to its broad-spectrum antimicrobial activity and wound healing potential [133,134]. Traditionally, honey has been used to treat various wounds, including burns, ulcers, diabetic wounds, and surgical injuries, due to its ability to inhibit microbial growth and facilitate tissue repair [133,135]. Bioactive compounds in honey, such as flavonoids, phenolic acids, organic acids, enzymes, and vitamins, work synergistically to enhance antioxidant defenses, reduce inflammation, and promote tissue regeneration [136,137]. Electron paramagnetic resonance (EPR) spectroscopy studies have elucidated the generation and scavenging of free radicals in different honey types for wound healing. For instance, commercial heat-processed honey typically lacks antibacterial activity, whereas pasture honeys exhibit antibacterial effects despite minimal peroxide production. Manuka honey is well-known for its strong antibacterial properties independent of peroxide generation [138,139]. The capacity of honey to neutralize ROS correlates with its antioxidant potential, with manuka honey rapidly scavenging free radicals within minutes [139,140]. Peroxide-producing honeys generate free radicals upon dilution, contributing to antimicrobial activity, while some honey types also help resolve chronic inflammation characteristics of non-healing wounds by modulating ROS production and quenching, thereby reducing oxidative stress and supporting tissue recovery [141,142]. Figure 6 presents summarized mechanisms of antimicrobial and wound healing effects of honey.

## 9. Food Processing

Honey from various floral sources has gained considerable attention as a functional ingredient in the food industry due to its natural antioxidant properties, sweetening capacity, and potential to replace synthetic additives. Recent studies have evaluated its application in salad dressings, meat preservation, and food formulations, where oxidative stability and sensory quality are critical [144,145]. Rasmussen et al. [144] conducted a comprehensive evaluation using clover and blueberry honeys, selected based on their high oxygen radical absorbance capacity (ORAC) values and phenolic compound profiles identified through high-performance liquid chromatography (HPLC). These honeys were incorporated into a French salad dressing system and subjected to storage under accelerated oxidative conditions at 37 °C for six weeks, and at ambient (23 °C) and refrigerated (4 °C) conditions for one year. The study found that salad dressings containing honey exhibited oxidation resistance comparable to that provided by EDTA, a synthetic chelator widely used in commercial formulations to inhibit lipid oxidation. Further supporting the antioxidative role of honey, McKibben and Engeseth [145] evaluated its effectiveness in mitigating lipid peroxidation in ground turkey. Using thiobarbituric acid reactive substances (TBARS) as a marker of oxidation, they showed that acacia honey at 5% (*w/w*) reduced TBARS levels by approximately 34%, while buckwheat honey at the same concentration reduced it by nearly 70% after three days of storage at 4 °C. These reductions were significantly greater than those observed with tocopherol and butylated hydroxytoluene (BHT), both commonly used antioxidants in meat products. Such findings underline honey’s dual role as a natural preservative and healthier sweetener alternative, supporting its potential to replace high fructose corn syrup (HFCS) and synthetic antioxidants in food processing. Beyond meat and dressings, the antioxidant potential of different honeys has been quantified using assays such as the antioxidant capacity of water-soluble (ACW) and lipid-soluble (ACL) substances, showing wide applicability in lipid-rich food and functional formulations [146,147]. In a broader context, replacing synthetic additives with honey aligns with the consumer demand for “clean label” products and supports food industry efforts to reduce exposure to chemical preservatives while maintaining oxidative stability and sensory quality. Moreover, since honey contains a complex mixture of flavonoids, phenolic acids, enzymes, and organic acids, its antioxidant function may also confer additional health benefits beyond shelf-life extension, such as protection against oxidized lipids and related inflammatory conditions [148].

A summary of the wide range of therapeutic properties of honey is presented in Table 2.

## 10. Anti-Inflammatory and Immunological Properties of Honey

Honey has demonstrated significant anti-inflammatory and immunomodulatory effects, primarily attributed to its rich content of flavonoids, phenolic acids, and other bioactive compounds. These molecules modulate immune responses through a variety of mechanisms, including the downregulation of pro-inflammatory cytokines, suppression of oxidative stress pathways, and modulation of intracellular signaling cascades such as NF-κB and MAPKs. In vitro and in vivo studies have consistently shown that honey inhibits the production of key pro-inflammatory mediators such as tumor necrosis factor-alpha (TNF-α), interleukin-1 beta (IL-1β), and interleukin-6 (IL-6), which play central roles in the initiation and propagation of inflammation. For instance, it is demonstrated that manuka honey significantly reduced levels of inflammatory cytokines and oxidative stress markers in a rat model of inflammation, suggesting its potential as a natural anti-inflammatory agent [149]. Caffeic acid phenethyl ester (CAPE), a bioactive component found in some honey types, has been particularly well studied for its immunomodulatory effects. CAPE suppresses the nuclear translocation of NF-κB, thereby downregulating inflammatory gene expression and inhibiting the release of cytokines such as IL-2 and IFN-γ from T-cells [150,151]. Similarly, chrysin, another honey-derived flavonoid, was found to inhibit lipopolysaccharide (LPS)-induced production of nitric oxide (NO) and cytokines in macrophages by interfering with NF-κB and Akt signaling pathways [152]. Moreover, honey has shown promise in enhancing immune responses during infection and tissue damage. In animal models, honey treatment resulted in increased proliferation of lymphocytes and enhanced phagocytic activity of immune cells [153]. The immunostimulatory effects may also be partially attributed to trace amounts of honeybee-derived peptides and enzymes such as defensin-1 and glucose oxidase, which contribute to antimicrobial defense while modulating host immunity [132]. The ability of honey to balance immune function, suppressing excessive inflammation while supporting innate immune defense, makes it particularly attractive in the context of chronic inflammatory diseases and wound healing. Table 3 summarizes the antioxidant activities of different honey types.

## 11. Conclusions

Honey’s antioxidant capacity arises from its rich and diverse bioactive compounds. Key antioxidant compounds not only exhibit strong free radical scavenging abilities but also modulate various cellular and molecular pathways relevant to health and disease. The neuroprotective properties of flavonoids like apigenin and pinocembrin underscore honey’s potential in mitigating neurodegenerative disorders by attenuating oxidative stress, neuroinflammation, and apoptosis. Moreover, honey’s antimicrobial activities are broad-spectrum, and these properties render honey an effective natural agent against pathogens involved in wound infections and food spoilage. Advanced applications, including tissue-engineered scaffolds incorporating honey, are expanding their clinical utility in regenerative medicine. While the therapeutic potential of honey is well-supported by numerous studies, several important limitations must be considered when interpreting these findings. A major challenge lies in the compositional variability of honey, which depends heavily on botanical origin, geographical region, and pre-/post-harvest processing practices. This variability affects the concentration and profile of bioactive compounds, leading to significant inter-study heterogeneity and limiting reproducibility across both experimental and clinical studies. Moreover, batch-to-batch consistency is seldom reported or controlled for in research, further complicating efforts to compare findings or formulate standardized therapeutic products. Although honey has a lower glycemic index than refined sugars, it raises concerns about its glycemic load, particularly in diabetic or insulin-resistant consumers. Additionally, dosing inconsistencies are apparent across the literature, with studies employing a wide range of dosages, from small dietary quantities to high pharmacological concentrations, without standardized guidelines or reference ranges. In summary, the multifaceted antioxidant, neuroprotective, antimicrobial, wound healing, immunological, anti-inflammatory, and food preservative properties of honey establish it as a valuable natural product with significant potential in both health-related and industrial applications. Mentioned limitations underscore the need for continued research into its bioactive compounds and mechanisms of action, which will further elucidate honey’s role in disease prevention and therapy, as well as in sustainable food preservation strategies.

## Figures and Tables

**Table 1:** Summary of the main constituents of honey and their functions.

Components Group	Examples	Concentration Range	Function/Relevance
flavonoids	quercetin, kaempferol, myricetin, chrysin, pinobanksin, pinocembrin, hesperetin	trace–varies by floral source	antioxidant, anti-inflammatory, antimicrobial
phenolic acids	gallic acid, caffeic acid, p-coumaric acid, ferulic acid, vanillic acid, syringic acid, chlorogenic acid, ellagic acid, 4-hydroxybenzoic acid	trace–high variability	antioxidant, antimicrobial, and chemotaxonomic markers
volatile organic compounds	linalool, lilac aldehyde/alcohol isomers, benzaldehyde, 2-phenylethanol, acetic acid, furfural, isophorone, vomifoliol, dmhbme	trace (<0.1%)	aroma, flavor, antimicrobial, and antioxidant activity
organic acids	gluconic acid (dominant), acetic acid, formic acid, citric acid	0.57–1.0%	acidity, preservation, antimicrobial, taste
sugars	fructose (38–40%), glucose (31–35%), maltose (7–10%), sucrose (1–5%)	~79.6–80% total sugars	energy source, sweetness, osmotic preservation, Maillard reaction, antioxidants
proteins and enzymes	glucose oxidase, diastase, invertase, catalase, albumins	0.266–0.50%	antimicrobial (via hydrogen peroxide), wound healing, and digestion of sugars
amino acids	proline (dominant), glutamic acid, aspartic acid, phenylalanine	0.05–0.1%	nutritional value, Maillard reaction precursors
vitamins	vitamin C, B-complex (B1, B2, B3, B5, B6, B9), vitamin k, vitamin e	trace–0.5% total	antioxidant, metabolic cofactor, immune support
minerals and trace elements	potassium, calcium, iron, magnesium, zinc, manganese, selenium	0.04–0.17%	enzymatic cofactors, antioxidant defense (e.g., Se, Zn)
other phytochemicals	carotenoids, sugar alcohols, alkaloids, tannins	trace	antioxidant, immune modulation
colloids and nitrogenous compounds	colloidal proteins, peptides, nitrogenous bases	~0.043% nitrogen compounds	structural and biological activity enhancers

Note: Given concentrations are approximate and highly variable depending on botanical source, climatic conditions, and processing management.

**Table 2:** Summary of therapeutic properties of honey.

Type of Study	Honey Type/Compound	Therapeutic Area	Key Findings/Effects	Mechanism/Activity	References
clinical trial	multifloral honey	wound healing	enhanced healing of diabetic ulcers	antimicrobial, anti-inflammatory	[25]
clinical and in vitro	buckwheat honey	antioxidant	increased serum antioxidant capacity; protected endothelial cells	ROS inhibition, GSH restoration, lipid peroxidation prevention	[58,59,60]
in vitro	quercetin, caffeine, phenethyl ester	anticancer (antiangiogenic)	inhibited tube formation, endothelial proliferation	suppression of angiogenesis	[65]
in vitro	quercetin, flavonoids	anticancer	enhanced p53 and fas-ligand expression in tumor cells	induced apoptosis	[76]
animal model	honey + caffeine	anticancer	reduced colon tumorigenesis	synergistic inhibition of tumor growth	[70]
in vitro	chrysin	anticancer (glioma, leukemia)	triggered apoptosis, p21 activation, caspase-3 activation	p38-MAPK, proteasome inhibition	[72,73,74]
	galangin	anticancer	induced selective DNA fragmentation in HL-60 cells	apoptotic signaling	[64]
review	honey polyphenols	cancer, sleep regulation	emphasized the multifunctionality of polyphenols	multi-targeted pathways	[69]
	various honeys	antimicrobial	effective against bacteria and fungi	enzymatic activity, H_2_O_2_, low pH, phenolics	[53]
clinical study	raw honey	gastrointestinal health	relieved gastritis symptoms	antibacterial, mucosal protection	[56,57]
animal study	Iranian honey	anti-inflammatory/neuroprotective	reduced neuroinflammation markers	cytokine suppression, antioxidant enzymes	[53]
animal model	tualang honey	antidiabetic cardiovascular	lowered blood glucose, improved lipid profile	antioxidant, insulin sensitivity	[47,48,49]
in vitro	gelam honey	antioxidant	strong free radical scavenging	high flavonoid content	[53]
	manuka honey	antibacterial	inhibited *S. aureus*, *E. coli* growth	MGO content, H_2_O_2_ release	[51,52]
	various honey types	antioxidant/food processing	High ORAC values; effective in lipid stabilization	polyphenol content	[53,54]
clinical study	honey vs. corn syrup	cardiovascular antioxidant support	improved levels of vitamin C, β-carotene, uric acid, GSH	serum antioxidant response	[25,60]
biochemical	button sage and buckwheat	food/antioxidant quality	high antioxidant equivalents (21.3 × 10^−5^; 4.32 × 10^−3^)	phenolic marker analysis	[54,55]
analytical	various honeys	bioavailability	phenolics detectable in plasma after consumption	confirmed absorption	[61]
clinical	processed honeys	food/health maintenance	still elevated plasma antioxidant levels	synergistic polyphenol interaction	[62]
reviews	honey phytochemicals	oncology	emphasized chemopreventive relevance	multi-pathway targeting	[67,68]
in vitro/in vivo	CAPE, chrysin, pinocembrin	anti-inflammatory immunomodulatory	reduced pro-inflammatory cytokines, enhanced immune response	NF-κB inhibition, MAPK modulation, caspase activation	[71,72,73,74]

**Table 3:** Comparative antioxidant activity of different types of honey and methods used for evaluation.

Honey Type	Country/Floral Source	Antioxidant Method(s)	Total Antioxidant Activity/Results	References
manuka honey	New Zealand/*Leptospermum scoparium*	DPPH, FRAP, ORAC	high antioxidant activity; strong radical scavenging in DPPH assay	[149]
tualang honey	Malaysia/wild rainforest	DPPH, FRAP	comparable to vitamin C in scavenging activity; high phenolic content	[150]
buckwheat honey	USA, Europe/*Fagopyrum* spp.	DPPH, ORAC	one of the highest antioxidant capacities; similar to 1 mM α-tocopherol	[151,152]
gelam honey	Malaysia/*Melaleuca cajuputi*	FRAP, ABTS	high reducing power and flavonoid concentration	[153]
heather honey	Europe/*Calluna vulgaris*	DPPH, FRAP	high total phenolic content; strong metal chelating activity	[154]
acacia honey	Europe/*Robinia pseudoacacia*	DPPH, FRAP	low to moderate antioxidant activity due to lower polyphenol levels	[155]
sidr honey	Yemen/*Ziziphus spina-christi*	DPPH, FRAP	moderate activity; antioxidant potential varies by region	[156]
eucalyptus honey	Mediterranean/*Eucalyptus* spp.	DPPH, FRAP, TAC	rich in phenolic acids; moderate to high activity	[157]
multifloral honey	various regions	DPPH, FRAP	highly variable depending on floral origin and processing	[158]
